# The mediating role of psychological empowerment in the relationship between job crafting and employee performance perception

**DOI:** 10.3389/fpsyg.2026.1792640

**Published:** 2026-05-11

**Authors:** Evin Miser

**Affiliations:** Department of Social Security, Ankara University, Ayaş Vocational School, Ankara, Türkiye

**Keywords:** job crafting, performance, information technologies, psychological empowerment, task performance, contextual performance

## Abstract

**Introduction:**

Employee performance is a key indicator of organizational effectiveness. One important factor shaping employee performance is job design. This study examines the relationship between job crafting—a bottom-up approach to job design that has received increasing attention in recent years—and employees’ perceptions of performance, as well as the mediating role of psychological empowerment in this relationship.

**Methods:**

Using a cross-sectional design, survey data were collected from 403 participants in the information technology (IT) sector, where creativity and innovation are core characteristics of the work. Five-point Likert scales were used for the Job Crafting Scale, Psychological Empowerment Scale, and Performance Scale. Reliability and validity were supported. The proposed research model was analyzed using structural equation modeling (SEM).

**Results:**

Job crafting was significantly associated with contextual performance (β≈0.80, *p* < 0.001) and task performance (β≈0.80, *p* < 0.001). It was also positively associated with psychological empowerment (β≈0.47, *p* < 0.001). However, psychological empowerment was not significantly associated with either performance dimension, and the indirect effects were non-significant (confidence intervals included zero).

**Discussion:**

The findings revealed positive, statistically significant relationships among job crafting, contextual performance, task performance, and psychological empowerment. However, the hypotheses that psychological empowerment mediates the relationship between job crafting and performance were not supported. Results indicate that proactive, bottom-up work design can improve performance independently of empowerment mechanisms, potentially reflecting autonomy and dynamic demands in IT work.

## 1 Introduction

Job design is a key determinant of employee productivity and organizational effectiveness, focusing on how work is organized and how employees perform in their daily work (Oldham and Fried, 2016). In the early 2000s, significant changes in technology, the emergence of new job types, the integration of information technologies into production processes, the growing dominance of the service sector, the increasing heterogeneity of the workforce, the abundance of attractive job opportunities, and the progressive deregulation of labor markets reshaped the landscape of work design (Hornung et al., 2011). Within this context, major models and approaches in job design theory were revisited and debated. According to Oldham and Hackman (2010), the traditional meaning of the term “job” has evolved because the very phenomena being studied are constantly changing.

Contemporary perspectives emphasize that jobs are no longer static; consequently, employees’ new roles require greater adaptability and self-determination (Wrzesniewski and Dutton, 2001). This dynamic shift renders traditional, purely top-down organizational job redesign initiatives insufficient for improving motivation and performance (Grant et al., 2011).

Within this dynamic view, attention has shifted from top-down redesign to employee-initiated adjustments. One of the most important approaches that has received increasing attention in this context is job crafting, a bottom-up approach to job design. Job crafting refers to the proactive physical and cognitive changes individuals make in the task or relational boundaries of their work (Wrzesniewski and Dutton, 2001). Tims and Bakker (2010), on the other hand, added a new dimension to job crafting from a Job Demands-Resources (JD-R) perspective. Within this framework, these proactive adjustments involve aligning job demands and resources with personal goals. The literature includes numerous studies highlighting the positive outcomes of job crafting. Notable results include job satisfaction (Slemp and Vella-Brodrick, 2013), job engagement (Tims and Bakker, 2010; Petrou et al., 2012; Chen et al., 2014), performance (Chen et al., 2014), innovative behavior (Bakker et al., 2012), and positive psychological capital (Slemp et al., 2015). In particular, job crafting that increases resources and challenges has been linked to extra-role contributions, conceptually aligned with contextual performance, highlighting job crafting’s relevance for day-to-day functioning in modern workplaces (Demerouti and Bakker, 2014).

A closely related psychosocial construct in this context is psychological empowerment. Conceptualized as a motivational state characterized by meaning, competence, self-determination, and impact, empowerment explains how individuals embrace their work and enhance their performance (Spreitzer, 1995). The core idea of empowerment in the workplace involves decentralizing authority across all organizational levels and supporting employees in taking initiative in strategic decision-making (Conger and Kanungo, 1988; Kanter, 1983). Although empowerment has multiple conceptual traditions, meta-analytic evidence indicates that it is consistently associated with important organizational outcomes and employee functioning and is shaped by both contextual work characteristics and individual factors (Seibert et al., 2011). More recent quantitative reviews similarly reinforce empowerment’s relevance across outcomes and highlight its role as a psychological mechanism through which work experiences may translate into motivation and performance (Llorente-Alonso et al., 2023). Because job crafting often involves seeking autonomy, resources, and meaningfulness, empowerment has been proposed as a plausible psychological mechanism through which proactive work experiences may translate into higher motivation and performance.

Understanding how these proactive behaviors and empowerment levels relate to performance is critical. Modern frameworks describe individual work performance as multidimensional, commonly distinguishing task performance (core job execution and fixed duties) from contextual performance (voluntary behaviors that support the social and psychological environment of the organization). However, despite growing interest, evidence remains mixed on whether psychological empowerment fully explains (i.e., mediates) the relationship between job crafting and these performance outcomes.

Accordingly, this study examines the relationships among job crafting, psychological empowerment, and employees’ perceptions of performance. Although prior research has examined these constructs, evidence on their interrelationships remains inconclusive. To address this gap, our study contributes to the literature in three significant ways: First, this study re-examines the mediating role of psychological empowerment in the relationship between job crafting and employees’ perceived performance. Second, in response to Oldham and Hackman’s (2010) call for future research to adapt to changing work phenomena, it explores the psychological mechanisms underlying proactive work behaviors from an empowerment perspective. Third, by focusing exclusively on professionals in the information technology (IT) sector, a demographic characterized by high levels of education, flexibility, and creativity, the current research offers a more detailed examination of how proactive job design operates within an evolving work environment and a changing employee profile.

## 2 Literature review

### 2.1 Job crafting

A notable development in the job design literature was the introduction of job crafting by Wrzesniewski and Dutton (2001) in the early 2000s. Job crafting, broadly defined as the process by which employees actively shape their own jobs to better align with their needs, values, skills, and abilities, emerged, as previously noted, in response to profound changes in the nature and context of work. In traditional job design approaches, management was primarily responsible for designing jobs, while employees merely carried out the work as structured by management (Oldham and Fried, 2016). This approach is referred to as top-down job design. In contrast, following Wrzesniewski and Dutton’s (2001) work, job crafting has been conceptualized as a bottom-up approach to job design, emphasizing employees’ active roles in shaping and redefining their work experiences.

According to Wrzesniewski and Dutton (2001), job crafting refers to the physical and cognitive changes individuals make in the task or relational boundaries of their work. In other words, it involves employees proactively reshaping the tasks they perform, the relationships they build, and the meaning they ascribe to their work to better align with their personal preferences, values, and strengths. In this regard, job crafting can be considered an innovative and improvisational process (Sözber and Ergeneli, 2019).

Tims et al. (2012) conceptualized job crafting within the Job Demands-Resources (JD-R) model. Job crafting is defined as the self-initiated changes that employees make in their own job demands and job resources to attain and/or optimize their personal (work) goals (Tims et al., 2012, p. 173). The study identified four independent dimensions of job crafting: increasing structural job resources, increasing social job resources, increasing challenging job demands, and decreasing hindering job demands. *Increasing structural job resources* refers to employees’ efforts to enhance their job resources, such as seeking opportunities for professional development, gaining greater autonomy, or improving their skills. *Increasing social job resources* involves employees’ attempts to obtain social support in the work environment, for example, seeking support from supervisors, help from coworkers, or performance feedback. Within the Job Demands-Resources (JD-R) model, job demands/requirements are conceptualized in two distinct forms. *Increasing challenging job demands* encompasses efforts to take on tasks that foster a sense of accomplishment, promote career growth, and encourage engagement, such as volunteering for new projects or pursuing opportunities for personal development. Finally, *decreasing hindering job demands* refers to employees’ proactive attempts to reduce stressors that impede performance. Employees are expected to identify and mitigate physical or psychological factors that hinder their job performance and productivity (Bakker et al., 2016).

The literature includes numerous studies highlighting the positive outcomes of these proactive behaviors. Notable results include job satisfaction (Slemp and Vella-Brodrick, 2013), job engagement (Petrou et al., 2012), innovative behavior (Bakker et al., 2012), and overall performance (Chen et al., 2014). In particular, job crafting that increases resources and challenges has been linked to extra-role contributions, which are conceptually aligned with contextual performance, highlighting job crafting’s relevance to day-to-day functioning in modern workplaces (Demerouti and Bakker, 2014).

Previous studies have examined both the antecedents and outcomes of job crafting. According to Wrzesniewski and Dutton (2001), job crafting is a proactive, improvisational process driven primarily by employees’ basic psychological needs for personal control, positive self-image, and human connection. Employees’ levels of job autonomy and decision latitude serve as perceived opportunities that facilitate this behavior. The positive relationship between decision latitude and job crafting has been confirmed in other studies (Leana et al., 2009; Lyons, 2008). For example, Lyons (2008) found positive associations between self-image, perceived control, readiness for change, and job crafting. Similarly, from a JD-R perspective, Tims et al. (2012) argue that a proactive personality trait increases employees’ demand for structural and social job resources and encourages them to pursue challenging job demands, thereby positioning proactive personality and individual initiative as antecedents of job crafting, while cynicism is negatively associated with it.

These proactive adaptations to the work environment consistently foster job engagement and lead to improved performance (Chen et al., 2014; Petrou et al., 2012). Among the benefits of job crafting is employees’ enhanced job satisfaction, which in turn makes their work more meaningful (Wrzesniewski and Dutton, 2001). Another positive outcome, benefiting both employees directly and organizations indirectly, is improved performance (Leana et al., 2009; Ghitulescu, 2007). In particular, job crafting that increases resources and challenges has been linked to extra-role contributions, conceptually aligned with contextual performance, highlighting its relevance for day-to-day functioning in modern workplaces (Demerouti and Bakker, 2014). Furthermore, the positive relationships between job crafting and wellbeing (Tims et al., 2012) as well as psychological empowerment (Miller, 2015) are particularly noteworthy.

### 2.2 Psychological empowerment

A closely related psychosocial construct in this context is psychological empowerment. Conceptualized as a motivational state characterized by meaning, competence, self-determination, and impact, psychological empowerment explains how individuals embrace their work and enhance their performance (Spreitzer, 1995). The core idea of empowerment in the workplace is to decentralize authority across all organizational levels and to support employees in taking initiative in strategic decision-making (Conger and Kanungo, 1988; Kanter, 1983).

Among studies examining the antecedents of psychological empowerment, there is a general consensus that the work climate, shaped by organizational structure, management policies, and practices, is a key antecedent (Chen et al., 2007; Wallace et al., 2011; Seibert et al., 2011; Maynard et al., 2012; Liden et al., 2000). In addition to individual antecedents such as self-efficacy (Bandura, 1997), intrinsic motivation (Thomas and Velthouse, 1990), locus of control (Spreitzer, 1995), and proactive or open personality traits (Seibert et al., 2011), organizational antecedents include empowering leadership (Ahearne et al., 2005; Sosik et al., 2014), organizational justice (Niehoff and Moorman, 1993), participatory decision-making (Kanter, 1983), and open access to strategic information (Conger and Kanungo, 1988).

Contemporary studies examining how employee empowerment practices generate both individual and organizational benefits have yielded valuable findings. Through the mediating roles of meaning and impact at work, higher job satisfaction is associated with psychological empowerment (Ahmad and Oranye, 2010; Pelit et al., 2011; Namasivayam et al., 2014). Another frequently identified individual outcome is that empowerment enhances organizational commitment (Liden et al., 2000; Ambad and Bahron, 2012; Raub and Robert, 2013; Kimura, 2011). Scholars have also demonstrated that psychological empowerment is positively related to creativity and innovative motivation (Zhang and Bartol, 2010; Hebenstreit, 2012; Knol and van Linge, 2009), lower turnover intentions (Kim and Fernandez, 2017), reduced burnout (Laschinger et al., 2011; Demir Uslu and Çavuş, 2010), and greater psychological wellbeing (Spreitzer, 2012). At the organizational level, studies have shown that psychological empowerment contributes to innovation (Ahearne et al., 2005) and team performance (Kirkman and Rosen, 1999). Moreover, empowered employees are more likely to engage in organizational citizenship behaviors (Chiang and Hsieh, 2012).

In recent years, an increasing number of studies have examined the relationship between job crafting and psychological empowerment. This relationship has attracted growing scholarly attention because the antecedents, benefits, and outcomes of both constructs are closely intertwined. Han and Jeong (2025) demonstrated that psychological empowerment is a critical mechanism that fosters job crafting behaviors among nurses.

Similarly, Piao and Hahn (2025) found that empowering leadership stimulates job crafting at the team level by enhancing psychological empowerment. Wang et al. (2025) examined the chain mediation of psychological empowerment and job crafting, showing that psychological empowerment enhances employees’ job crafting behaviors, which, in turn, positively influence job satisfaction and wellbeing. Zhang et al. (2025) proposed a conceptual model suggesting a reciprocal relationship between empowerment and job crafting. Tims and Bakker (2010) emphasized the intrinsic role of empowerment in their Job Demands-Resources (JD-R) model, while Petrou et al. (2012) argued that job crafting strengthens the impact of empowerment on job satisfaction.

### 2.3 Performance

One of the most important indicators of organizational success is employee performance. According to Motowidlo (2003), job performance is the total value expected from the voluntary behavioral acts exhibited by an individual within an organization over a specific period. The first key element in this definition is the behavioral nature of performance; it consists of voluntary, sustained behaviors that persist over time. Second, this set of behaviors must reflect the organization’s expectations and generate added value. Therefore, the behaviors exhibited by different individuals should not be confused with those exhibited by the same individual across different time periods. This distinction is based on “the extent to which employees contribute to the effectiveness of the organization” (Motowidlo, 2003).

Performance is one of the ultimate outcomes organizations seek to achieve. Consequently, the conditions and factors that enhance performance have been the focus of numerous studies in the literature. Diverse conceptualizations of performance have led to different approaches to defining its dimensions. Among these, the most widely recognized framework distinguishes two main dimensions: task performance and contextual performance (Kart, 2015).

*Task performance* refers to the fixed duties and responsibilities associated with a job (Jawahar and Carr, 2007). It encompasses behaviors that support the technical foundations of job descriptions and enable the production of goods or services, thereby directly contributing to the organization’s technical requirements. Task performance is closely related to professional competence, a supportive work environment, a clear job description, and adherence to ethical standards (Özdevecioğlu and Kanıgür, 2009).

*Contextual performance*, a value-added component of overall organizational performance, has attracted increasing attention in recent years. Employees can influence organizational effectiveness through behaviors not directly tied to their formal duties. These behaviors shape the organizational, social, and psychological context in which work is performed (Goodman and Svyantek, 1999). Although these patterns have been labeled volunteering (Zeithaml et al., 1990) or organizational citizenship behaviors (Bateman and Organ, 1983; Smith et al., 1983), the present study uses the term contextual performance, originally coined by Borman and Motowidlo (1993). Examples include complying with organizational rules, even when they are not explicitly stated; helping colleagues; supporting organizational goals; completing others’ tasks when necessary (Goodman and Svyantek, 1999); and maintaining courteous and positive workplace relationships. According to Borman and Motowidlo (1993), all activities that support and ensure the success of organizational work constitute contextual performance. These behaviors, although not formally included in employees’ job descriptions, contribute to the social and psychological environment of the organization and significantly affect the achievement of desired overall performance objectives (Polatçı, 2011).

## 3 Research model and hypotheses development

### 3.1 Job crafting and performance

Using the Job Demands-Resources (JD-R) model, proactive behaviors such as job crafting help optimize resources and challenges, naturally boosting employee motivation and performance. Based on this framework, the following hypotheses are proposed:

*H1*: Job crafting is positively associated with contextual performance.

*H2*: Job crafting is positively associated with task performance.

### 3.2 Job crafting and psychological empowerment

Because job crafting often involves seeking autonomy, resources, and meaningfulness, it naturally fosters employees’ sense of competence and impact. Studies suggest that these bottom-up, proactive behaviors are positively associated with employees’ psychological empowerment (Miller, 2015). Thus, the following hypothesis is proposed:

*H3*: Job crafting is positively associated with psychological empowerment.

### 3.3 The mediating role of psychological empowerment

Theoretically, psychological empowerment is a critical cognitive mechanism that translates proactive work behaviors into tangible outcomes. Drawing on the integration of the JD-R model and empowerment theory, employees who proactively optimize their job resources and demands (job crafting) experience a heightened sense of meaning, competence, autonomy, and impact. This internal motivational state, in turn, drives them to invest more effort in both their core duties (task performance) and voluntary extra-role behaviors (contextual performance). Although previous empirical studies have supported empowerment as a mediator in various contexts (e.g., Farzaneh et al., 2014; Kim and Kim, 2013), our primary theoretical premise is that job crafting structurally cultivates empowerment, which subsequently fosters performance. Thus, the following hypotheses are proposed:

*H4*: Psychological empowerment mediates the relationship between job crafting and contextual performance.

*H5*: Psychological empowerment mediates the relationship between job crafting and task performance.

## 4 Materials and methods

In this study, a quantitative, non-experimental, cross-sectional research design with a deductive approach was adopted to address the research questions and test the theoretically derived hypotheses (Trochim and Donnelly, 2001). Consistent with the previous literature, a quantitative methodology was deemed the most appropriate for thoroughly understanding the relationships among the variables. Furthermore, Structural Equation Modeling (SEM) was selected as the primary analytical technique. SEM was preferred because the foundation of this research relies on understanding a complex network of relationships among multiple variables, integrating confirmatory factor analysis, path analysis, and multiple regression within a single framework.

### 4.1 Sampling and data collection

This study examines the mediating role of psychological empowerment in the relationship between job crafting and employees’ perceived performance. The information technology (IT) sector was selected as the research context for its inherent creativity, innovation, and flexibility. Data were collected from 403 professionals employed by information and communication technology (ICT) companies located within technology development zones in Ankara, Türkiye.

Participants completed the survey either electronically or on paper. Before data collection, permission to use the measurement tools was obtained from the respective authors, and ethical approval was granted by the Institutional Ethics Committee of Ankara University (Protocol no: 167/9, Date: 07.12.2020). All participants were informed of the study’s purpose, assured of data confidentiality, and provided voluntary informed consent. The survey instrument also included items to capture participants’ demographic characteristics.

To assess sample size adequacy, an a priori power analysis in G*Power initially indicated a minimum of 102 participants. However, because G*Power is primarily designed for general multivariate analyses and may not fully capture the complexity of structural equation modeling (SEM), additional estimates were conducted. Monte Carlo-based power simulations in R and Stata indicated that a sample of at least 300 participants would provide adequate statistical power for the SEM analyses. The final sample of 403 valid responses comfortably exceeded this threshold, accommodating potential outliers and ensuring robust model estimation.

### 4.2 Measurement tools

#### 4.2.1 Job crafting

Job crafting was measured using the 21-item scale developed by Tims et al. (2012). The scale comprises four sub-dimensions: increasing structural job resources (α = 0.82), decreasing hindering job demands (α = 0.79), increasing social job resources (α = 0.77), and increasing challenging job demands (α = 0.75). Akın et al. (2014), adapted the scale into Turkish and reported an overall Cronbach’s alpha of 0.84 for the adapted version.

#### 4.2.2 Psychological empowerment

Psychological empowerment was measured with the 12-item scale proposed by Spreitzer (1995). The scale comprises four sub-dimensions: meaning, autonomy, competence, and impact. The reliability of the Turkish adaptation has been demonstrated in prior studies, with reported Cronbach’s alpha coefficients of 0.72 (Sürvegil et al., 2013) and 0.89 (Altındiş and Özutku, 2011).

#### 4.2.3 Performance

Performance, the dependent variable in the research model, was measured with the 25-item scale originally developed by Goodman and Svyantek (1999) (α = 0.86). The instrument includes two sub-dimensions: contextual and task performance. Polatçı (2011) conducted the Turkish adaptation of the scale. In her pilot study, Polatçı simplified the original scale through exploratory factor analysis and removed six items, resulting in a 19-item Turkish version. The Cronbach’s alpha for this adapted scale was reported as 0.93 (Polatçı, 2011).

All scale items were rated on a five-point Likert-type scale ranging from 1 (“Strongly disagree”) to 5 (“Strongly agree”).

### 4.3 Data analysis

Preliminary analyses were conducted on a dataset from 403 participants who completed the questionnaire, which included relevant scales and demographic questions. Missing values were imputed, and outliers were removed. Consequently, the analyses were conducted using data from 314 participants. Reverse-coded items were recoded before analysis.

## 5 Results

This section presents the results of SEM-based statistical analyses used to test the research hypotheses. Both the measurement and structural models were evaluated to assess reliability, validity, and overall model fit.

### 5.1 Descriptive statistics and correlations

As shown in Table 1, the sample is predominantly male (63.4%), with female participants accounting for 36.3%. Regarding education, 65.9% of respondents hold a bachelor’s degree, while 28.6% possess postgraduate degrees (master’s or doctorate). This indicates that the vast majority of the sample (94.5%) consists of highly educated professionals, consistent with the characteristics of the IT sector. In terms of organizational positions, 41.4% hold administrative roles, 34.7% occupy technical roles, and 23.9% work in other capacities (such as legal, health, or academic roles within the technoparks). Furthermore, respondents demonstrate substantial professional experience, with over 55% having more than 10 years of total work experience. The detailed demographic characteristics of the participants are summarized in Table 1.

**TABLE 1:** Demographic profile of the participants.

Demographic variables	Category	Frequency (n)	Percentage (%)
Gender	Female	114	36.3
	Male	199	63.4
	Prefer not to say	1	0.3
Total		314	100.0
Education level	High school	17	5.4
	Bachelor’s degree	207	65.9
	Master’s degree	73	23.2
	Doctorate (PhD)	17	5.4
Total		314	100.0
Position/role	Administrative	130	41.4
	Technical	109	34.7
	Other	75	23.9
Total		314	100.0
Tenure at current firm	1–4 years	151	48.1
	5–9 years	67	21.3
	10–14 years	47	15.0
	15 years and above	47	15.0
	Missing	2	0.6
Total		314	100.0
Total work experience	1–4 years	60	19.1
	5–9 years	80	25.5
	10–14 years	74	23.6
Total		314	100.0

Tests of normality, a prerequisite for multivariate parametric analyses, were conducted. The results indicated that the dataset met the assumptions of multivariate normality and was therefore suitable for further analysis. Pearson correlation analysis was also conducted, and the results showed no evidence of multicollinearity among the study variables (Table 2).

**TABLE 2:** Means, standard deviations, Skewness, Kurtosis and correlations of all variables.

Variable	Mean	SD	Skewness	Kurtosis	Correlations coefficients		
					JC	PE	*P*
Job crafting (JC)	3.67	0.43	−0.181	1.017	1.000	0.298	0.367
Psychological empowerment (PE)	4.03	0.55	−0.666	1.677		1.000	0.270
Performance (P)	4.07	0.48	−0.777	1.311			1.000

### 5.2 Measurement model assessment

In SEM analyses, the reliability and validity of the measurement model were assessed using Cronbach’s alpha, composite reliability (CR), and average variance extracted (AVE) (Çark and Maşrap, 2019; Dinçer, 2020). Cronbach’s alpha and CR values from 0.60 to 0.70 indicate acceptable reliability, whereas values from 0.70 to 0.95 reflect high internal consistency (Hair et al., 2016). Moreover, AVE values greater than 0.50 indicate that the construct explains more than half of the variance in its indicators, thereby demonstrating adequate convergent validity (Fornell and Larcker, 1981). The Cronbach’s alpha, CR, and AVE values for the constructs in this study are presented in Table 3. The results indicate that the model exhibits high internal consistency, reliability, and convergent validity.

**TABLE 3:** Measurement model: Cronbach’s α, composite reliability (CR), average variance extracted (AVE), maximum shared variance (MSV), and average shared variance (ASV).

Variables	α	CR	AVE	MSV	ASV
Job crafting	0.820				
Increasing structural job resources	0.869	0.873	0.592	0.224	0.112
Decreasing hindering job demands	0.779	0.788	0.472	0.016	0.005
Increasing social job resources	0.794	0.797	0.441	0.232	0.042
Increasing challenging job demands	0.816	0.821	0.480	0.238	0.088
Psychological empowerment	0.878				
Competence	0.832	0.835	0.628	0.272	0.077
Meaning	0.800	0.801	0.574	0.158	0.080
Autonomy	0.870	0.871	0.694	0.227	0.087
Impact	0.859	0.860	0.672	0.220	0.098
Performance	0.924				
Contextual performance	0.910	0.911	0.485	0.224	0.065
Task performance	0.876	0.881	0.350	0.272	0.096

α, Cronbach’s alpha; CR, Composite Reliability; AVE, Average Variance Extracted; MSV, Maximum Shared Variance; ASV, Average Shared Variance. Values of CR > 0.70 and AVE > 0.50 indicate acceptable reliability and convergent validity (Hair et al., 2016). For divergent validity, MSV < AVE and ASV < MSV criteria were considered (Yaşlıoğlu, 2017; Sürücü et al., 2021).

Discriminant validity was assessed using the Maximum Shared Variance (MSV) and Average Shared Variance (ASV). Following the recommendations of Yaşlıoğlu (2017) and Sürücü et al. (2021, p. 2704), discriminant validity is supported when both MSV and ASV are lower than the Average Variance Extracted (AVE) for each construct (i.e., MSV < AVE and ASV < AVE). As shown in Table 3, all MSV and ASV values in this study were lower than the corresponding AVE values, indicating adequate discriminant validity.

### 5.3 Factor analysis

First-order confirmatory factor analysis (CFA) was conducted using maximum-likelihood estimation to assess the validity of the measurement scales for job crafting, psychological empowerment, and performance. Because the performance scale comprises only two sub-dimensions (contextual and task performance), modeling it as a higher-order latent variable could yield zero or negative degrees of freedom. Therefore, following Byrne’s (2016) recommendations, contextual and task performance were treated as two separate latent variables in the research model, whereas second-order CFAs were conducted for all other multidimensional constructs.

The first-order CFA results indicated acceptable model fit across the measurement tools. The chi-square-to-degrees-of-freedom ratio (χ^2^/df) was below the threshold of 5, and the Comparative Fit Index (CFI), Tucker-Lewis Index (TLI), Goodness-of-Fit (GFI), and Root Mean Square Error of Approximation (RMSEA) fell within the recommended ranges reported in the literature (Hair et al., 2016; Yaşlıoğlu, 2017). Thus, the results confirmed that the first-order CFA models demonstrated satisfactory convergent validity. The fit indices for the first-order CFA are presented in Table 4.

**TABLE 4:** First-order CFA results.

Variables	χ ^2^(df)	χ ^2^/df	CFI	TLI	GFI	RMSEA
Job crafting	278.9 (149)	1.87	0.946	0.938	0.913	0.053
Psychological empowerment	86.10 (29)	2.97	0.965	0.947	0.963	0.064
Contextual performance	120.3 (64)	1.88	0.952	0.943	0.914	0.058
Task performance	109.7 (60)	1.83	0.955	0.947	0.870	0.080

χ^2^, chi-square; df, degrees of freedom; CFI, comparative fit index; TLI, Tucker–Lewis index; GFI, goodness-of-fit index; RMSEA, root mean square error of approximation.

After validating the individual measures with a first-order CFA, a second-order CFA was estimated to assess the overall measurement model (performance was retained as two correlated first-order factors). The second-order measurement model showed adequate fit: χ^2^(1016) = 1667.1, *p* < 0.001; χ^2^/df = 1.64; CFI = 0.909; TLI = 0.903; GFI = 0.817; and RMSEA = 0.045.

These results meet the acceptance criteria proposed by Sürücü et al. (2021) and Byrne (2016). Unstandardized estimates, standardized factor loadings, and critical ratios (CRs) are presented in Table 5.

**TABLE 5:** Second-order CFA standardized loadings.

Variables	b (Unstd)	β (Std.)	CR (z)	*p*
Job crafting				
Increasing structural job resources	1 (fixed)	0.629		
Decreasing hindering job demands	0.091	0.071	0.960	0.337
Increasing social job resources	0.444	0.377	4.403	< 0.001
Increasing challenging job demands	0.0647	0.696	6.782	< 0.001
Psychological empowerment				
Competence	1 (fixed)	0.665		< 0.001
Meaning	1.150	0.767	9.884	< 0.001
Autonomy	1.385	0.834	10.744	< 0.001
Impact	1.578	0.921	11.452	< 0.001

b, unstandardized loading; β, standardized loading; CR (z), unstandardized estimate/standard error. “1 (fixed)” denotes the reference loading set for model identification.

During the evaluation of standardized loadings, the Decreasing Hindering Job Demands dimension did not load significantly on the higher-order job crafting construct (β = 0.071, *p* = 0.337). This finding aligns with prior research suggesting that reducing hindering demands is a defensive rather than a proactive job crafting strategy (Bakker et al., 2016; Tims et al., 2012). In this study, the results may also reflect occupational and cultural characteristics. Employees in the IT sector, who typically face dynamic work demands and value continuous learning, are more likely to engage in resource-enhancing or challenge-seeking behaviors than to reduce job demands. Moreover, in collectivist work environments like Türkiye, efforts to minimize workload or avoid tasks may be perceived less favorably and thus may not align with the proactive nature of job crafting. Consequently, the Decreasing Hindering Job Demands dimension was excluded from the subsequent structural model to ensure model parsimony and theoretical consistency.

### 5.4 Structural model and hypothesis testing

Path analysis using structural equation modeling (SEM) was conducted to test the research hypotheses and to assess the direction and strength of relationships among latent variables. Standardized path coefficients and hypothesis test results are presented in Tables 6, 7.

**TABLE 6:** Direct effects from the structural model with latent variables.

Hypothesis	Structural path	*b*	β	CR (z)	*p*	Result
H1	Job crafting → Contextual performance	0.990	0.801	6.757	< 0.001***	Supported
H2	Job crafting → Task performance	0.761	0.801	6.801	< 0.001***	Supported
H3	Job crafting → Psy. empowerment	0.421	0.468	4.760	< 0.001***	Supported
	Psy. empowerment → Task performance	−0.138	−0.101	−1.233	0.218	Not supported
	Psy. empowerment → Contextual performance	−0.015	0.014	−0.182	0.856	Not supported

*b*, unstandardized coefficient; β, standardized coefficient; CR (z), b/SE. ***p < 0.001.

**TABLE 7:** Direct and indirect (mediated) effects (bootstrapped, 5,000 resamples).

Hypothesis	Indirect path	Indirect β (95% CI)	Direct β	Total β (95% CI)	*p*	Result
H4	Job Crafting → Psy. emp. → Contextual performance	−0.06 (-0.31 to 0.03)	0.99	0.93 (0.71–1.34)	0.182	Not supported
H5	Job crafting → Psy. Emp. → Task performance	0.01 (-0.21 to 0.09)	0.76	0.77 (0.50–1.21)	0.846	Not supported

Bias-corrected 95% confidence intervals; bootstrap = 5,000 resamples.

The results indicate that job crafting has a significant positive direct effect on contextual performance (β = 0.801, *p* < 0.001). This finding supports H1, suggesting that employees who engage more actively in job crafting behaviors tend to demonstrate higher levels of contextual performance. Similarly, job crafting exerts a significant positive direct effect on task performance (β = 0.801, *p* < 0.001), confirming H2. Thus, higher levels of job crafting are associated with improved task performance outcomes. In addition, job crafting is significantly associated with psychological empowerment (β = 0.468, *p* < 0.001), supporting H3. This result implies that as employees engage in job crafting behaviors, their sense of autonomy, meaning, competence, and impact in their work also increases. Overall, the path analysis results provide empirical support for the proposed model, demonstrating that job crafting plays a central role in enhancing both psychological empowerment and employee performance dimensions.

To test the mediating role of psychological empowerment in the relationship between job crafting and performance outcomes (H4 and H5), a bootstrapping procedure with 5,000 resamples was used to compute 95% bias-corrected confidence intervals (CIs) for the indirect effects.

**FIGURE 1:**
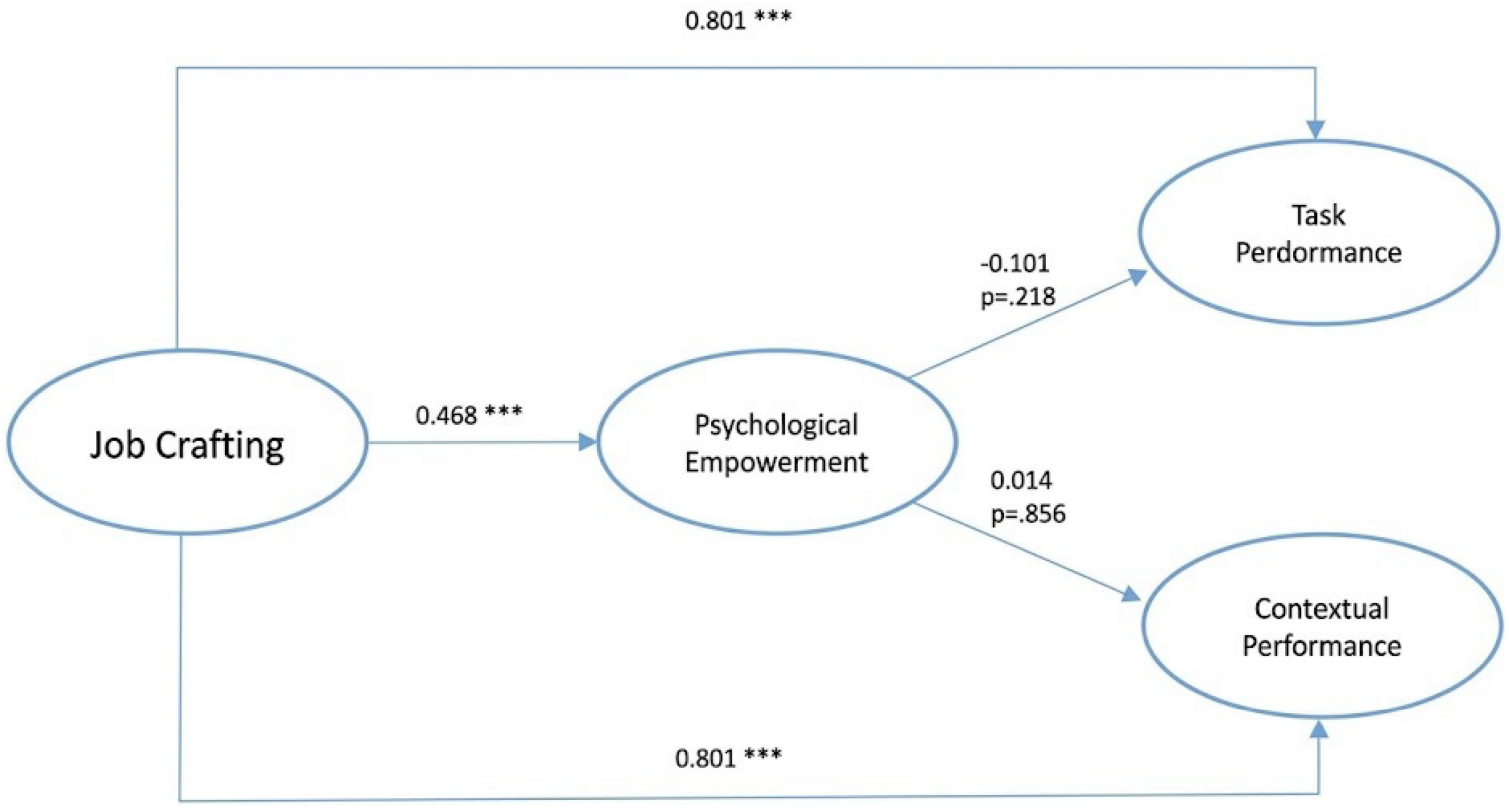
Structural path estimates model. All path estimates are standardized. ***p < 0.001.

Although job crafting was significantly associated with psychological empowerment, psychological empowerment was not significantly associated with either task performance (β = −0.101, *p* = 0.218) or contextual performance (β = 0.014, *p* = 0.856). Bootstrapped indirect effects further confirmed this non-significance, with indirect standardized effects of −0.06 (95% CI [−0.31, 0.03]) for contextual performance and 0.01 (95% CI [−0.21, 0.09]) for task performance. Because the confidence intervals for the indirect effects include zero, psychological empowerment does not significantly mediate the relationship between job crafting and either dimension of performance. Therefore, H4 and H5 are not supported. The direct, indirect, and total effects are summarized in Table 7 and illustrated in the structural model (Figure 1).

## 6 Discussion

The primary objective of this study was to examine the relationship between job crafting and employee performance, with psychological empowerment as a potential mediator. The results showed that job crafting was positively associated with both contextual and task performance, key indicators of organizational effectiveness (β = 0.80, *p* < 0.001 for contextual performance; β = 0.80, *p* < 0.001 for task performance). These findings suggest that in organizations that actively support job crafting, employees not only perform their formal job duties more effectively but also exhibit higher levels of discretionary, context-building behaviors. This outcome reinforces the importance of autonomy and proactive work design practices in fostering performance-oriented organizational climates.

It is also worth noting that the job crafting approach is more strongly associated with overall performance than with task performance, particularly when compared to job design models in which managerial influence predominates (Miser, 2022). This finding aligns with the notion that job crafting, by encouraging employees to shape their work relationships and construct personal meaning in their tasks, promotes behaviors that extend beyond formal job descriptions. Furthermore, the duties, authorities, and responsibilities assigned to employees by management are just as important as the discretionary behaviors that contribute to the social and psychological context of the organization, even if they are not explicitly included in job descriptions. The findings indicate that employees who experience autonomy in determining how their tasks are performed, with whom they collaborate, and how they interpret the meaning and purpose of their work—in other words, those who embody the characteristics of job crafting—tend to engage in behaviors that, although not formally defined, enhance organizational effectiveness. Moreover, employees with such profiles also perceive significantly higher performance in their defined roles.

Consistent with prior research (Spreitzer, 1995; Tims et al., 2012), job crafting was significantly associated with psychological empowerment (β = 0.47, *p* < 0.001), confirming its motivational role. However, contrary to expectations, psychological empowerment did not mediate the relationship between job crafting and either contextual or task performance. The indirect effects of job crafting through empowerment were statistically insignificant. This result suggests that the motivational benefits of job crafting may not necessarily operate through perceived empowerment. In the IT sector, where employees are typically characterized by high autonomy, adaptability, and technical self-efficacy, the influence of job crafting on performance may be more direct, stemming from engagement, creativity, and intrinsic interest rather than empowerment itself. In other words, employees confident in their capabilities may not require additional empowering mechanisms to sustain high performance. This interpretation aligns with studies proposing that the psychological outcomes of job crafting can, in certain contexts, substitute for rather than depend on empowerment processes (Kim and Kim, 2013; Farzaneh et al., 2014). Research findings also indicate that a job crafting strategy may inherently support employees’ psychological empowerment. Employees who can manage job challenges and increase the resources required for their work appear to need psychological empowerment less to achieve performance goals. Nevertheless, this association clearly needs to be tested through future research.

### 6.1 Limitations and future research

A primary limitation of this study is its cross-sectional design, which limits the ability to draw definitive causal inferences about the variables. Furthermore, reliance on self-reported data may introduce common method bias. Future studies should employ longitudinal or multi-source designs to mitigate these risks and better capture the dynamic nature of job crafting over time.

The current study is limited to private organizations in the information and communication technologies (IT) sector in Ankara. Future research could include employees from other sectors, such as healthcare, education, or industrial production, to broaden perspective and improve the generalizability of the findings.

This research used quantitative methods. Future studies could incorporate qualitative approaches to gain deeper insight into employees’ and managers’ perceptions of job design and job crafting.

From a theoretical standpoint, future research could also further investigate the Decreasing Hindering Job Demands (DHD) dimension, particularly its distinctiveness from other positively related job crafting dimensions.

## 7 Conclusion

As mentioned earlier, structural transformations in the labor market have made it difficult to revise existing job design models or develop new ones. Initiating job design by employees means they are no longer passive recipients of work processes but rather primary actors who actively shape their work environment. Indeed, it is now critical for employees to be at the forefront and play a central role in many job characteristics (Hornung et al., 2011). Furthermore, environmental factors influence the expected role of employees much more than previously assumed (Fried et al., 2007; Johns, 2006). Thus, the traditional management-centered approach to job design is being replaced by a job crafting approach, which transforms it into an employee-centered model. This demonstrates that the job crafting approach has the potential to support management strategies that can adapt to the structural transformation of modern working life.

## Data Availability

The raw data supporting the conclusions of this article will be made available by the author, without undue reservation.

## References

[B1] Ahearne M. Mathieu J. Rapp A. (2005). To empower or not to empower your sales force? *J. Appl. Psychol.* 90 945–955. 10.1037/0021-9010.90.5.945 16162066

[B2] Ahmad N. Oranye N. O. (2010). Empowerment, job satisfaction and organizational commitment: A comparative analysis of nurses working in Malaysia and England. *J. Nurs. Manag.* 18 582–591. 10.1111/j.1365-2834.2010.01093 20636507

[B3] Akın A. Sarıçam H. Kaya Ç Demir T. (2014). Ýş Becerikliliği Ölçeği’nin Türkçe versiyonu: Geçerlik ve güvenirlik çalışması. *Int. J. Educ. Res.* 5 10–15. Turkish

[B4] Altındiş S. Özutku H. (2011). Psikolojik güçlendirme ve güçlendirmeyi etkileyen faktörler: Türkiye’deki devlet hastanelerinde bir araştırma. *Afyon Kocatepe Üniversitesi Sosyal Bilimler Enstitüsü Dergisi* 13 161–191. Turkish

[B5] Ambad S. N. Bahron A. (2012). Psychological empowerment: The influence on organizational commitment among employees in the construction sector. *J. Glob. Bus. Manag.* 8 73–81.

[B6] Bakker A. B. Rodríguez-Muñoz A. Vergel A. I. S. (2016). Modelling job crafting behaviours: Implication for work engagement. *Hum. Rel.* 69 169–189. 10.1177/0018726715581690

[B7] Bakker A. B. Tims M. Derks D. (2012). Proactive personality and job performance: The role of job crafting and work engagement. *Hum. Rel.* 65 1359–1378. 10.1177/0018726712453471

[B8] Bandura A. (1997). *Self-efficacy: The Exercise of Control.* New York, NY: W. H. Freeman and Company.

[B9] Bateman T. S. Organ D. W. (1983). Job satisfaction and the good soldier: The relationship between affect and employee citizenship. *Acad. Manage. J.* 26 587–595. 10.2307/255908

[B10] Borman W. C. Motowidlo S. J. (1993). “Expanding the Criterion Domain to Include Elements of Contextual Performance,” in *Personnel Selection in Organizations*, eds Schmitt N. Borman W. C. (San Francisco, CA: Jossey–Bass), 71–98.

[B11] Byrne B. M. (2016). *Structural Equation Modeling with AMOS: Basic Concepts, Applications, and Programming*, 3rd Edn. New York, NY: Routledge.

[B12] Çark Ö Maşrap A. (2019). Kurumsal kaynak planlama kullanıcıları açısından sistemin faydalarını etkileyen faktörler. *3. Sektör Sosyal Ekonomi Dergisi* 54 992–1013. Turkish. 10.15659/3.sektor-sosyal-ekonomi.19.05.1127

[B13] Chen C.-Y. Yen C.-H. Tsai F. C. (2014). Job crafting and job engagement: The mediating role of person–job fit. *Int. J. Hosp. Manag.* 37 21–28. 10.1016/j.ijhm.2013.10.006

[B14] Chen Z. Lam W. Zhong J. A. (2007). Leader–member exchange and member performance: A new look at individual-level negative feedback-seeking behavior and team-level empowerment climate. *J Appl. Psychol.* 92 202–212. 10.1037/0021-9010.92.1.202 17227161

[B15] Chiang C.-F. Hsieh T.-S. (2012). The impacts of perceived organizational support and psychological empowerment on job performance: The mediating effects of organizational citizenship behavior. *Int. J. Hosp. Manag.* 31 180–190. 10.1016/j.ijhm.2011.04.011

[B16] Conger J. A. Kanungo R. N. (1988). The empowerment process: Integrating theory and practice. *Acad. Manage. Rev.* 13 471–482. 10.2307/258093 23842316

[B17] Demerouti E. Bakker A. B. (2014). “Job Crafting,” in *An Introduction to Contemporary Work Psychology*, eds Peeters, J. de Jonge M. C. W. Taris T. W. (Chichester: Wiley-Blackwell), 414–433.

[B18] Demir Uslu Y. Çavuş M. F. (2010). The impacts of structural and psychological empowerment on burnout: A research on staff nurses in Turkish state hospitals. *Can. Soc. Sci.* 6 63–72.

[B19] Dinçer E. (2020). *Örgüt kültürünün örgütsel bağlılık üzerine etkisi: Yetenek yönetiminin aracılık rolü.* PhD thesis, Türkiye: Ankara University, Social Sciences Institute. Turkish

[B20] Farzaneh J. Farashah A. D. Kazemi M. (2014). The impact of person–job fit and person–organization fit on OCB: The mediating and moderating effects of organizational commitment and psychological empowerment. *Pers. Rev.* 43 672–691. 10.1108/PR-07-2013-0118

[B21] Fornell C. Larcker D. F. (1981). Evaluating structural equation models with unobservable variables and measurement error. *J. Mark. Res.* 18 39–50. 10.1177/002224378101800104

[B22] Fried Y. Grant A. M. Levi A. S. Hadani M. Slowik L. H. (2007). Job design in temporal context: A career dynamics perspective. *J. Organ. Behav.* 28 911–927. 10.1002/job.486

[B23] Ghitulescu B. E. (2007). *Shaping Tasks and Relationships at Work: Examining the Antecedents and Consequences of Employee Job Crafting.* PhD thesis, Pittsburgh, PA: University of Pittsburgh.

[B24] Goodman S. A. Svyantek D. J. (1999). Person–organization fit and contextual performance: Do shared values matter? *J. Vocat. Behav.* 55 254–272. 10.1006/jvbe.1998.1682

[B25] Grant A. M. Fried Y. Juillerat T. (2011). “Work Matters: Job Design in Classic and Contemporary Perspectives,” in *APA Handbook of Industrial and Organizational Psychology: Building and Developing the Organization*, ed. Zedeck S. (Washington, DC: American Psychological Association), 417–453.

[B26] Hair J. F. Hult G. T. M. Ringle C. M. Sarstedt M. (2016). *A Primer on Partial Least Squares Structural Equation Modeling (PLS-SEM)*, 2nd Edn. Thousand Oaks, CA: Sage.

[B27] Han S. Jeong E. (2025). Development and validation of the clinical nurses’ job crafting scale. *J. Korean Acad. Nurs. Adm.* 31 17–28. 10.11111/jkana.2025.0017

[B28] Hebenstreit J. J. (2012). Nurse educator perceptions of structural empowerment and innovative behavior. *Nurs. Educ. Perspect.* 33 297–301. 23061186

[B29] Hornung S. Glaser J. Rousseau D. M. Angerer P. Weigl M. (2011). Employee-oriented leadership and quality of working life: Mediating roles of idiosyncratic deals. *Psychol. Rep.* 108 59–74. 10.2466/07.13.14.21.PR0.108.1.59-74 21526592

[B30] Jawahar I. M. Carr D. (2007). Conscientiousness and contextual performance: The compensatory effects of perceived organizational support and leader–member exchange. *J. Manag. Psychol.* 22 330–349. 10.1108/02683940710745923

[B31] Johns G. (2006). The essential impact of context on organizational behavior. *Acad. Manage. Rev.* 31 386–408.

[B32] Kanter R. M. (1983). *The Change Masters: Innovation for Productivity in the American Corporation.* New York, NY: Simon & Schuster.

[B33] Kart M. (2015). *Örgütsel sinizm.* Ankara: Nobel Akademik Yayıncılık. Turkish

[B34] Kim S. Y. Fernandez S. (2017). Employee empowerment and turnover intention in the U.S. federal bureaucracy. *Am. Rev. Public Adm.* 47 4–22. 10.1177/0275074015583712

[B35] Kim T.-Y. Kim M. (2013). Leaders’ moral competence and employee outcomes: The effects of psychological empowerment and person–supervisor fit. *J Bus. Ethics* 112 155–166. 10.1007/s10551-012-1238-1

[B36] Kimura T. (2011). Empowerment, P–O fit, and work engagement: A mediated moderation model. *Eur. J. Econ. Finance Adm. Sci.* 38 44–58.

[B37] Kirkman B. L. Rosen B. (1999). Beyond self-management: Antecedents and consequences of team empowerment. *Acad. Manage. J.* 42 58–74. 10.2307/256874 22761606 PMC3385647

[B38] Knol J. van Linge R. (2009). Innovative behaviour: The effect of structural and psychological empowerment on nurses. *J Adv. Nurs.* 65 359–370. 10.1111/j.1365-2648.2008.04876.x 19191936

[B39] Laschinger H. K. S. Wong C. A. Grau A. L. Read E. A. Pineau Stam L. M. (2011). The influence of leadership practices and empowerment on Canadian nurse manager outcomes. *J. Nurs. Manage.* 20 877–888. 10.1111/j.1365-2834.2011.01307.x 23050621

[B40] Leana C. Appelbaum E. Shevchuk I. (2009). Work process and quality of care in early childhood education: The role of job crafting. *Acad. Manage. J.* 52 1169–1192. 10.5465/AMJ.2009.47084651

[B41] Liden R. C. Wayne S. J. Sparrowe R. T. (2000). An examination of the mediating role of psychological empowerment on the relations between the job, interpersonal relationships, and work outcomes. *J. Appl. Psychol* 85 407–416. 10.1037/0021-9010.85.3.407 10900815

[B42] Llorente-Alonso M. García-Ael C. Topa G. (2023). A meta-analysis of psychological empowerment: Antecedents, organizational outcomes, and moderating variables. *Curr. Psychol.* 43 1–26. 10.1007/s12144-023-04369-8

[B43] Lyons P. (2008). The crafting of jobs and individual differences. *J Bus. Psychol.* 23 25–36. 10.1007/s10869-008-9080-2

[B44] Maynard M. T. Gilson L. L. Mathieu J. E. (2012). Empowerment - Fad or fab? A multilevel review of the past two decades of research. *J. Manage.* 38 1231–1281. 10.1177/0149206312438773

[B45] Miller M. (2015). *Relationships between Job Design, Job Crafting, Idiosyncratic Deals, and Psychological Empowerment.* PhD thesis, Minneapolis, MN: Walden University.

[B46] Miser E. (2022). *Ýş karakteristikleri, iş becerikliliği ve kişiye özgü anlaşmalar ile performans ilişkisinde psikolojik güçlendirmenin aracılık rolü.* PhD thesis, Türkiye: Ankara University, Social Sciences Institute. Turkish

[B47] Motowidlo S. J. (2003). “Job performance,” in *Handbook of Psychology: Industrial and Organizational Psychology*, eds Borman W. C. Ilgen D. R. Klimoski R. J. (Hoboken, NJ: John Wiley & Sons), 39–53.

[B48] Namasivayam K. Guchait P. Lei P. (2014). The influence of leader empowering behaviors and employee psychological empowerment on customer satisfaction. *Int. J. Contemp. Hosp. Manag.* 26 69–84. 10.1108/IJCHM-11-2012-0218

[B49] Niehoff B. P. Moorman R. H. (1993). Justice as a mediator of relationships between monitoring and citizenship. *Acad. Manage. J.* 36 527–556. 10.5465/256591

[B50] Oldham G. R. Fried Y. (2016). Job design research and theory: Past, present and future. *Organ. Behav. Hum. Decis. Process.* 136 20–35. 10.1016/j.obhdp.2016.05.002

[B51] Oldham G. R. Hackman J. R. (2010). Not what it was and not what it will be: The future of job design research. *J. Organ. Behav.* 31 463–479. 10.1002/job.678

[B52] Özdevecioğlu M. Kanıgür S. (2009). Çalışanların ilişki ve görev yönelimli liderlik algılamalarının performansları üzerindeki etkileri. *KMU ÝÝBF Dergisi* 16 53–82. Turkish

[B53] Pelit E. Öztürk Y. Arslantürk Y. (2011). The effects of employee empowerment on employee job satisfaction: A study on hotels in Turkey. *Int. J. Contemp. Hosp. Manag.* 23 784–802. 10.1108/09596111111160006

[B54] Petrou P. Demerouti E. Peeters M. C. Schaufeli W. B. Hetland J. (2012). Crafting a job on a daily basis: Contextual correlates and the link to work engagement. *J. Organ. Behav.* 33 1120–1141. 10.1002/job.1783

[B55] Piao J. Hahn J. (2025). How empowering leadership drives proactivity in the Chinese IT industry: Mediation through team job crafting and psychological safety with ICT knowledge as a moderator. *Behav. Sci.* 15:609. 10.3390/bs15050609 40426387 PMC12109525

[B56] Polatçı S. (2011). *Psikolojik sermayenin performans üzerindeki etkisinde iş–aile yayılımı ve psikolojik iyi oluşun rolü.* PhD thesis, Türkiye: Erciyes University, Social Sciences Institute. Turkish

[B57] Raub S. Robert C. (2013). Empowerment, organizational commitment, and voice behavior in the hospitality industry: Evidence from a multi-national sample. *Cornell Hosp. Q.* 54 136–148. 10.1177/1938965512465067

[B58] Seibert S. E. Wang G. Courtright S. H. (2011). Antecedents and consequences of psychological and team empowerment in organizations: A meta-analytic review. *J. Appl. Psychol.* 96 981–1003. 10.1037/a0022676 21443317

[B59] Slemp G. R. Vella-Brodrick D. A. (2013). The Job Crafting Questionnaire: A new scale to measure the extent to which employees engage in job crafting. *Int. J. Wellbeing* 3 126–146.

[B60] Slemp G. R. Kern M. L. Vella-Brodrick D. A. (2015). Workplace well-being: The role of job crafting and autonomy support. *Psychol. Well-Being* 5:7. 10.1186/s13612-015-0034-y

[B61] Smith C. A. Organ D. W. Near J. P. (1983). Organizational citizenship behavior: Its nature and antecedents. *J. Appl. Psychol.* 68 653–663. 10.1037/0021-9010.68.4.653

[B62] Sosik J. J. Chun J. U. Zhu W. (2014). Hang on to your ego: The moderating role of leader narcissism on relationships between leader charisma and follower psychological empowerment and moral identity. *J. Bus. Ethics* 120 65–80. 10.1007/s10551-013-1651-0

[B63] Sözber S. Ergeneli A. (2019). Dışsal prestijin, iş becerikliliğinin ve kişi-örgüt uyumunun iş–aile çatışması ile ilişkisi. *Ýşletme Araştırmaları Dergisi* 11 3404–3420. Turkish. 10.20491/isarder.2019.817

[B64] Spreitzer G. M. (1995). Psychological empowerment in the workplace: Dimensions, measurement, and validation. *Acad. Manage. J.* 38 1442–1465. 10.2307/256865

[B65] Spreitzer G. M. (2012). “Taking Stock: A Review of Psychological Empowerment,” in *The Oxford Handbook of Positive Organizational Scholarship*, eds Cameron K. S. Spreitzer G. M. (Oxford: Oxford University Press), 54–68. 10.1016/S0140-6736(25)01637-X

[B66] Sürücü L. Şeşen H. Maslakçı A. (2021). *SPSS, AMOS ve Process Macro ile ilişkisel, aracı/düzenleyici ve yapısal eşitlik modellemesi.* Ankara: Detay Yayıncılık. Turkish

[B67] Sürvegil O. Tolay E. Topoyan M. (2013). Yapısal güçlendirme ve psikolojik güçlendirme ölçeklerinin geçerlilik ve güvenirlik analizleri. *J Yaşar Univ.* 8 5371–5391. Turkish

[B68] Thomas K. W. Velthouse B. A. (1990). Cognitive elements of empowerment: An interpretive model of intrinsic task motivation. *Acad. Manage. Rev.* 15 666–681. 10.2307/258687

[B69] Tims M. Bakker A. B. (2010). Job crafting: Towards a new model of individual job redesign. *SA J. Ind. Psychol.* 36:841. 10.4102/sajip.v36i2.841

[B70] Tims M. Bakker A. B. Derks D. (2012). Development and validation of the Job Crafting Scale. *J. Vocat. Behav.* 80 173–186. 10.1016/j.jvb.2011.05.009

[B71] Trochim W. M. K. Donnelly J. P. (2001). *Research Methods Knowledge Base.* Cincinnati, OH: Atomic Dog Publishing.

[B72] Wallace J. C. Johnson P. D. Mathe K. Paul J. (2011). Structural and psychological empowerment climates, performance, and the moderating role of shared felt accountability: A managerial perspective. *J. Appl. Psychol.* 96 840–850. 10.1037/a0022227 21381808

[B73] Wang L. Qiao T. Wang X. Wang C. Ye P. (2025). The impact of work–family conflict on early childhood teachers’ occupational well-being: The chain mediating role of psychological empowerment and job crafting. *Front. Public Health* 12:1513514. 10.3389/fpubh.2024.1513514 39845653 PMC11753349

[B74] Wrzesniewski A. Dutton J. E. (2001). Crafting a job: Revisioning employees as active crafters of their work. *Acad. Manage. Rev.* 26 179–201.

[B75] Yaşlıoğlu M. M. (2017). Sosyal bilimlerde faktör analizi ve geçerlilik: Keşfedici ve doğrulayıcı faktör analizlerinin kullanılması. *Ýstanbul Üniversitesi Ýşletme Fakültesi Dergisi* 46 74–85. Turkish

[B76] Zeithaml A. A. Parasuraman A. Berry L. L. (1990). *Delivering Service Quality: Balancing Customer Perceptions and Expectations.* New York, NY: The Free Press.

[B77] Zhang X. M. Bartol K. M. (2010). Linking empowering leadership and employee creativity: The influence of psychological empowerment, intrinsic motivation, and creative process engagement. *Acad. Manage. J.* 53 107–128. 10.5465/amj.2010.48037118

[B78] Zhang X. Yu K. Li W.-D. Zacher H. (2025). Sustainability of passion for work? Change-related reciprocal relationships between passion and job crafting. *J. Manage.* 51 1349–1383. 10.1177/01492063231207343

